# Tracking intracellular dynamics through extracellular measurements

**DOI:** 10.1371/journal.pone.0205031

**Published:** 2018-10-17

**Authors:** Franz Hamilton, Tyrus Berry, Timothy Sauer

**Affiliations:** 1 North Carolina State University, Department of Mathematics, Raleigh, 27695, United States of America; 2 George Mason University, Department of Mathematical Sciences, Fairfax, 22030, United States of America; Georgia State University, UNITED STATES

## Abstract

Extracellular recordings of neuronal cells are frequently a part of *in vitro* and *in vivo* experimental studies as a means of monitoring network-level dynamics. Their connections to intracellular dynamics are not well understood. Single-unit recordings are a more direct way to measure intracellular dynamics, but are typically difficult and expensive. On the other hand, simple differential equations models exist for single neurons. In this article, we apply a recent advance in data assimilation theory, designed to correct bias in general observation functions, toward the reconstruction of model-based intracellular dynamics from extracellular recordings.

## Introduction

*In vitro* and *in vivo* neuronal experiments in the laboratory are frequently centered around measurements of cell potential. While intracellular (within cell) recordings give precise, single-cell measurements of neuronal potential, they are difficult, often obtained under nonphysiological conditions, and typically do not allow for multi-site recordings. Extracellular recordings are easier to obtain, but the relation to intracellular dynamics is complicated. In particular, the morphology of the spikes and other aspects of the recordings are usually quite different.

**Fig 1:**
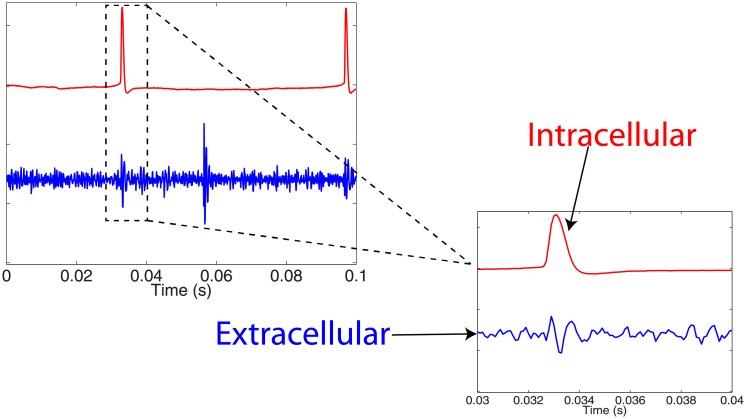
Example of simultaneous intracellular and extracellular recordings from a neuronal experiment in the laboratory. Data from [3–5]. Intracellular recordings (red trace) occur inside an individual cell, whereas extracellular recordings (blue trace) occur outside the cell and often result in the recording of activity from several different cells. While neuronal models describe the intracellular cell dynamics, experimental recordings often consist of extracellular measurements, particularly when examining network-level dynamics. There is a fundamental difference between the recorded potentials, for example the biphasic waveform of the extracellular recording compared to the monophasic waveform of the intracellular recording.

The recorded extracellular potential is in general a complicated sum of spatially distributed currents [1] within a complex extracellular space [2]. There is a complicated relationship between the spatial location of the extracellular measurement with respect to the cell, resulting in different waveform properties based on the distance from the recording site to the neuron. Fig 1 shows an example time series [3] comparing intracellular and extracellular recordings (see [4, 5] for more details on the experimental design and study). This representative example demonstrates some of the differences between the two types of cell measurements.

Modern methods of data assimilation are adept at reconstructing hidden variables, as long as details of their relations to observations are known. If we consider the intracellular dynamics as the hidden variable, it is the lack of an explicit “observation function” that has limited the applicability of these methods. While ad hoc methods for pre-processing the extracellular measurements have been used (see for example [6, 7]), these approaches suffer from never learning the true mapping from the intracellular state to the extracellular observation space.

In this article we assume as given an extracellular recording and a model of single-cell neuronal dynamics. We will exploit a recent advance [8] in data assimilation to fit the recording to the individual cell dynamics, in absence of a known relation between them. The recent advance shows how to learn state-dependent observation function bias while filtering a signal. The approach is general enough to be used with a wide range of data assimilation methods, including nonlinear methods such as the ensemble Kalman filter. The main point is that even though the exact observation function of the single-cell dynamics that relates its activity to the extracellular recording is unknown, the connection can be gradually learned from an iterative processing of the data.

Several works have examined the error caused by bias resulting from model mismatch, when the true system dynamics are not known [9–11], and when the actual observation function which maps the model state to observation space is unknown [8, 12]. Our work here essentially addresses both of these problems simultaneously within the context of neuronal intracellular state reconstruction. Not only do we lack an accurate observational model, but we must also reconcile the model error resulting from our use of a simplified dynamical model to assimilate complex extracellular measurements from a population of neurons.

In the following, we will show that intracellular dynamics reconstruction can occur even though a simple, generic neuron model is used as part of the data assimilation procedure. We begin, as proof of concept, by showing the successful reconstruction of the intracellular state given extracellular observations of a stochastic Fitzhugh-Nagumo cell. To further simulate the model error we will encounter when analyzing experimental data, we consider the assimilation of extracellular data from a more sophisticated model, the Hodgkin-Huxley system. Finally, we demonstrate the utility of our method in recovering the intracellular dynamics in an experimental setting by assimilating *in vivo* extracellular recordings.

## Methods

### The filtering problem

In the general filtering problem, we assume a system with *n*-dimensional state vector *x* and *m*-dimensional observation vector *y* defined in discrete time by

$$x_{k}=f\left(x_{k-1}\right)+w_{k-1}$$ (1)

$$y_{k}=h\left(x_{k}\right)+v_{k}$$ (2)

where *w*_*k*−1_ and *v*_*k*_ are white noise processes with covariance matrices *Q* and *R*, respectively. *f* represents the system dynamics and *h* is an observation function that maps the model state to the observation space. The goal is to sequentially estimate the state of the system given some noisy observations.

In the case of linear system dynamics and linear observation function, the Kalman filter [13] gives the optimal estimate of the system state. Extensions of the Kalman filter to nonlinear situations include the extended Kalman filter and ensemble-based Kalman filters [14–24], which approximate nonlinear systems and gives near-optimal estimates. In either linear or nonlinear situation, the assumption is that both *f* and *h* are known, allowing the filter to alternate between a forecast and analysis step. During the filter’s forecast, a model-based prediction of the system state and covariance is made. The predicted state is then mapped to observation space through known observation function *h*, giving a model-predicted observation. These state and covariance predictions are updated in the analysis step, which relies on the difference between the actual observation and the model predicted observation. This procedure continues iteratively for each observation.

Without loss of generality, here we consider use of the ensemble Kalman filter (EnKF) for nonlinear state estimation. The EnKF approximates a nonlinear system by forming an ensemble, such as through the unscented transformation (see for example [14]). At the *k*th step of the filter there is an estimate of the state $x_{k-1}^{+}$ and the covariance matrix $P_{k-1}^{+}$. In the unscented version of the EnKF, the singular value decomposition is used to find the symmetric positive definite square root $S_{k-1}^{+}$ of the matrix $P_{k-1}^{+}$, allowing us to form an ensemble of *E* state vectors where the *i*^*th*^ ensemble member is denoted $x_{i,k-1}^{+}$.

The model *f* is applied to the ensemble, and then observed with function *h*

$$\begin{array}{ccc} x_{i,k}^{-} & = & f(x_{i,k-1}^{+})\\ y_{i,k}^{-} & = & h(x_{i,k}^{-}) \end{array}$$ (3)

The mean of the resulting state and observed ensembles gives the prior state estimate $x_{k}^{-}$ and predicted observation $y_{k}^{-}$, respectively. Denoting the covariance matrices $P_{k}^{-}$ and $P_{k}^{y}$ of the resulting state and observed ensemble, and the cross-covariance matrix $P_{k}^{xy}$, we define

$$\begin{array}{ccc} P_{k}^{-} & = & \frac{1}{E}\sum_{i=1}^{E}(x_{i,k}^{-}-x_{k}^{-})(x_{i,k}^{-}-x_{k}^{-}{)}^{T}+Q\\ P_{k}^{y} & = & \frac{1}{E}\sum_{i=1}^{E}(y_{i,k}^{-}-y_{k}^{-})(y_{i,k}^{-}-y_{k}^{-}{)}^{T}+R\\ P_{k}^{xy} & = & \frac{1}{E}\sum_{i=1}^{E}(x_{i,k}^{-}-x_{k}^{-})(y_{i,k}^{-}-y_{k}^{-}{)}^{T} \end{array}$$ (4)

and use the equations

$$\begin{array}{ccc} K_{k} & = & P_{k}^{xy}{\left(P_{k}^{y}\right)}^{-1}\\ P_{k}^{+} & = & P_{k}^{-}-K_{k}P_{k}^{yx}\\ x_{k}^{+} & = & x_{k}^{-}+K_{k}(y_{k}-y_{k}^{-}). \end{array}$$ (5)

to update the state and covariance estimates with the observation *y*_*k*_. *Q* and *R* are generally unknown quantities that have to be estimated. We use the method of [25] for the adaptive estimation of these noise covariance matrices.

### Filtering with an unknown observation function

The update of the state and covariance predictions in the analysis step is dependent on the correct observation function *h* being known. Of course in many applications, such as in the mapping of intracellular model state to extracellular observation space, this function is known imperfectly and in its place an incorrect function *g* is used. We will assume the true dynamics are represented by Eqs 1 and 2, but that the filter is supplied with Eq 1 and

$$y_{k}=g\left(x_{k}\right)+v_{k}.$$ (6)

Define *b*(*x*_*k*_) = *h*(*x*_*k*_) − *g*(*x*_*k*_) to be the state-dependent *bias* in the observation resulting from use of the incorrect observation function. Recent work [12] addressed the issue of error caused by an unknown observation function by using a training set consisting of observations and the corresponding true state with which to build an estimate of the bias. Here, we assume that no training data of the true state are available and implement a recent advance [8] in data assimilation that attempts to empirically estimate the bias. We present a summary of the technique here, further details of which can be found in [8].

The general idea is to iteratively update the incorrect observation function *g* by obtaining improved estimates of the bias. We begin with an initial estimate of the bias function *b*^(0)^ = 0, and set *g*^(0)^ = *g* + *b*^(0)^ = *g*. The filter is given the known system dynamics *f*, the initial incorrect observation function *g*^(0)^, and the observations *y*, and provides an estimate of the state at each observation time *k*, which we denote $x_{k}^{\left(0\right)}$. This initial state estimate will be subject to large errors, due to the unaccounted-for bias. Using this imperfect state estimate, we calculate a noisy estimate of the bias, ${\hat{b}}_{k}^{\left(0\right)}$, corresponding to observation *y*_*k*_ where

$${\hat{b}}_{k}^{\left(0\right)}=y_{k}-g(x_{k}^{\left(0\right)}).$$ (7)

Due to noise in the data as well as the imperfection of the state estimate, ${\hat{b}}_{k}^{\left(0\right)}$ will not accurately reflect the true bias, *b*(*x*_*k*_). To build a better estimate of *b*(*x*_*k*_), we use Takens’ method of attractor reconstruction [26–29] to reconstruct the bias function. Given observation *y*_*k*_, consider delay-coordinate vector *z*_*k*_ = [*y*_*k*_, *y*_*k*−1_, …, *y*_*k*−*d*_] where *d* is the number of delays. Delay vector *z*_*k*_ represents the state of the system [26, 28]. To build the reconstruction, we locate the *N* nearest neighbors (with respect to Euclidean distance) $z_{i_{1}},\ldots ,z_{i_{N}}$, where

$$z_{i_{j}}=\left[y_{i_{j}},y_{i_{j}-1},\ldots ,y_{i_{j}-d}\right]$$

within the set of observations. Once the neighbors are found, the corresponding ${\hat{b}}_{i_{1}}^{\left(0\right)},{\hat{b}}_{i_{2}}^{\left(0\right)},\ldots ,{\hat{b}}_{i_{N}}^{\left(0\right)}$ values are used in a locally constant model to estimate *b*(*x*_*k*_)

$$b^{\left(0\right)}\left(x_{k}\right)=w_{i_{1}}{\hat{b}}_{i_{1}}^{\left(0\right)}+w_{i_{2}}{\hat{b}}_{i_{2}}^{\left(0\right)}+\ldots +w_{i_{N}}{\hat{b}}_{i_{N}}^{\left(0\right)}.$$ (8)

where the weight for *j*^*th*^ neighbor is defined as

$$w_{i_{j}}=\frac{e^{-(d_{i_{j}}/\sigma )}}{\sum_{j=1}^{N}e^{-(d_{i_{j}}/\sigma )}}.$$

Here, $d_{i_{j}}$ is the distance of $z_{i_{j}}$ to the current delay-coordinate vector and *σ* is the bandwidth which controls the contribution of each neighbor in the local model. We set *σ* to be half of the mean distance of the neighbors.

Note that Eq 8 is still just an approximation of *b*(*x*_*k*_), although a more accurate estimate compared to Eq 7. The observation function can now be updated as

$$g^{\left(1\right)}=g+b^{\left(0\right)}.$$

This improved observation function is given to the filter, and the data are re-processed. An improved state estimate, $x_{k}^{\left(1\right)}$, at time *k* is obtained, a more accurate reconstruction, *b*^(1)^(*x*_*k*_), of the bias is formed using Eqs 7 and 8 and the observation function is updated, *g*^(2)^ = *g* + *b*^(1)^.

The method continues iteratively, each iteration an improved reconstruction of *b*(*x*_*k*_) is obtained resulting in a better estimate of the state on the next iteration. To summarize the method for ℓ=0,1,…,M iterations

1. Initialize *g*^(0)^ = *g* and *b*^(0)^ = 0
2. For each observation *y*_*k*_, use filter to estimate state $x_{k}^{\left(\ell \right)}$ given known *f* and observation function *g*^(*ℓ*)^
3. Calculate noisy bias estimate ${\hat{b}}_{k}^{\left(\ell \right)}=y_{k}-g\left(x_{k}^{\left(\ell \right)}\right)$
4. Use Takens’ method to reconstruct estimate of bias function, *b*^(*ℓ*)^(*x*_*k*_)
5. Update observation function, *g*^(*ℓ*+1)^ = *g* + *b*^(*ℓ*)^
6. Repeat steps 2-5 until convergence

**Fig 2:**
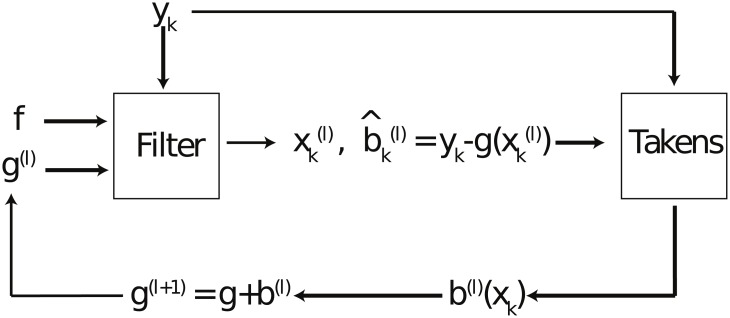
Schematic of algorithm for correcting observation error.

We determine that our method has converged when the change between successive reconstructions of the bias functions falls below some threshold. Fig 2 shows a schematic view of the steps of the algorithm.

## Results

In the results presented below, we assume noisy extracellular data are available from a neuronal system and we implement an EnKF with a generic intracellular spiking model to reconstruct the intracellular state of the system. Specifically, our EnKF is provided the Fitzhugh-Nagumo model [30, 31]

$$\begin{array}{cc} \dot{v} & =-w+v-\frac{v^{3}}{3}+I\\ \dot{w} & =\frac{v+0.7-0.8w}{\tau } \end{array}$$ (9)

where *I* is a known stimulating current and *τ* is a time-scale parameter that is used to adjust the spiking frequency of the model to that of the data. The variable *v* represents the intracellular potential and *w* is a recovery variable that lumps together the effects of different ionic currents.

Motivated by experimental scenarios, we will rely on the ansatz that measurements of the neuron extracellular potential are given by the negative time derivative of the intracellular potential. Thus give the EnKF is provided a “best guess”, though likely incorrect, observation function *g*

$$g\left(v,w\right)=-(-w+v-\frac{v^{3}}{3}+I)$$ (10)

which is just the negative right-hand side of the $\dot{v}$ differential equation in Eq 9.

Throughout, we will compare our bias corrected filter with the standard filter (essentially, the *ℓ* = 0 iteration of our method) which assumes no bias correction. Due to the extreme error caused by the biased observations, we limit the diagonal of the estimated noise covariances matrices to prevent overfitting or underfitting of the data.

### State estimation with an incorrect observation function

We consider the noise-driven Fitzhugh-Nagumo system

$$\begin{array}{cc} \dot{v} & =-w+v-\frac{v^{3}}{3}+I+I_{\text{noise}}\\ \dot{w} & =\frac{v+0.7-0.8w}{12.5} \end{array}$$ (11)

where $I=0.3\;\text{sin}(\frac{2\pi }{30}t)+0.1$ is a periodic forcing current and *I*_*noise*_ is a mean 0 Gaussian noise current with variance *σ*^2^ = 0.005. We assume that 2400 seconds of data are available, sampled at rate *dt* = 0.4. The true observation function

$$h\left(v,w\right)=\alpha_{1}f_{1}^{2}\left(v,w\right)+\alpha_{2}f_{1}\left(v,w\right)+\alpha_{3},$$ (12)

where $f_{1}\left(v,w\right)=-w+v-\frac{v^{3}}{3}+I+I_{noise}$, is unknown to us and in its place we give the EnKF the observation function described by Eq 10. The parameters *α*_1_, *α*_2_, *α*_3_ are used to control the amount of mismatch between the true observation function *h* and our filter observation function *g*, allowing us to test our algorithm at different error levels. Note that there is a degree of model error in this example since Eq 11 is stochastically driven whereas our assimilation model described by Eq 9 does not account for this additional noise term.

In our first example, we consider a situation where there is a small discrepancy between *h* and *g*. Specifically, we define parameters *α*_1_ = 0.1, *α*_2_ = −0.9, *α*_3_ = 0.01 in Eq 12 resulting in the observation function

$$h\left(v,w\right)=0.1f_{1}^{2}\left(v,w\right)-0.9f_{1}\left(v,w\right)+0.01.$$ (13)

**Fig 3:**
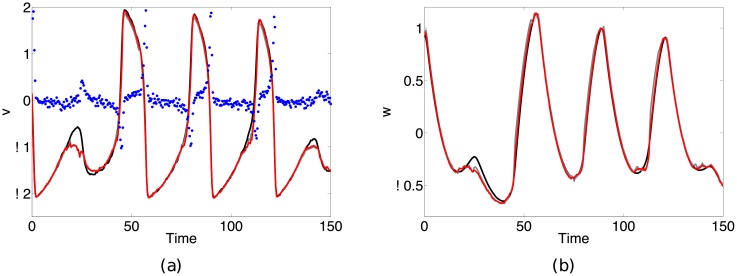
Results from reconstructing the state of a noise driven Fitzhugh-Nagumo system with small observation function error. Reconstruction results for the (a) intracellular potential *v* and (b) lump ionic recovery variable *w* are shown. The true observation function $h\left(v,w\right)=0.1f_{1}^{2}\left(v,w\right)-0.9f_{1}\left(v,w\right)+0.01$ is unknown to us, and in its place we use the observation model in Eq 10. Given the observations (blue circles), we attempt to reconstruct the true trajectories of *v* and *w* (solid black lines). In this example, the error between the filter observation function and true observation function is small enough that the standard filter (solid gray line) is able to provide accurate estimates of the variable trajectories (RMSE = 0.10, 0.07 for *v* and *w* respectively). The bias-corrected filter (solid red line) provides small, but not substantial improvements (RMSE = 0.10, 0.03 for *v* and *w* respectively).

Fig 3 shows the results of reconstructing *v* and *w* (black solid lines) given the observations (blue circles) resulting from the function in Eq 13. The solid gray line denotes the EnKF estimate without bias correction and the solid red line red line the estimate with bias correction. Note that the bias in this example is small, and as such the standard EnKF is able to handle the error and give good estimates of the system state by adjusting the *Q* and *R* covariance matrices (RMSE = 0.10, 0.07 for *v* and *w* respectively). The bias corrected filter, which uses *d* = 5 delays and *N* = 20 nearest neighbors to reconstruct the bias function, gives a slight improvement on the reconstruction of *w*, but in general performs comparably to the standard filter (RMSE = 0.10, 0.03 for *v* and *w* respectively). This result is not surprising, as we would not expect our bias-corrected filter to have much of an affect when the observational bias is low.

When the error in the observation function increases, the standard filter fails to accurately reconstruct the system dynamics. As an example, consider a large observation error scenario where *α*_1_ = 0.25, *α*_2_ = −0.85, *α*_3_ = 0.02 resulting in the observation function

$$h\left(v,w\right)=0.25f_{1}^{2}\left(v,w\right)-0.85f_{1}\left(v,w\right)+0.02.$$ (14)

**Fig 4:**
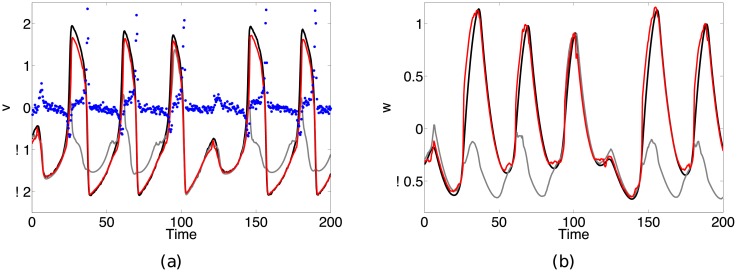
Results from reconstructing the state of a noise driven Fitzhugh-Nagumo system with large observation function error. Reconstruction results for the (a) *v* and (b) *w* system variables (solid black lines denote true trajectories) given the noisy observations (solid blue circles). The large error between the filter observation function in Eq 10 and the unknown true observation function, $h\left(v,w\right)=0.25f_{1}^{2}\left(v,w\right)-0.85f_{1}\left(v,w\right)+0.02$, causes the standard filter (solid gray line) to fail in tracking the system dynamics (RMSE = 0.95, 0.53 for *v* and *w* respectively). The bias-corrected filter (solid red line) significantly improves on the state reconstruction (RMSE = 0.26, 0.12 for *v* and *w* respectively).

Fig 4 shows the results of reconstructing the system state in this large bias situation. The filter with no bias correction (solid gray line) fails to track the dynamics of the variables (RMSE = 0.95, 0.53 for *v* and *w* respectively) while the bias-corrected filter (solid red line) is able to accurately reconstruct the system dynamics (RMSE = 0.26, 0.12 for *v* and *w* respectively). This significant improvement in state reconstruction highlights the capability of our method for reconciling large errors caused by observational mismatch.

### Reconstructing intracellular potential from extracellular measurements

The next example explores the assimilation of data from a Hodgkin-Huxley cell to a Fitzhugh-Nagumo model. The Hodgkin-Huxley system [32] is a detailed neuron model defined by

$$\begin{array}{ccc} C\dot{V} & = & -g_{1}m^{3}h\left(V-E_{1}\right)-g_{2}n^{4}\left(V-E_{2}\right)-g_{3}\left(V-E_{3}\right)+I_{\text{stim}}+I_{\text{noise}}\\ \dot{m} & = & \left(1-m\right)\alpha_{m}\left(V-E_{0}\right)-m\beta_{m}\left(V-E_{0}\right)\\ \dot{n} & = & \left(1-n\right)\alpha_{n}\left(V-E_{0}\right)-n\beta_{n}\left(V-E_{0}\right)\\ \dot{h} & = & \left(1-h\right)\alpha_{h}\left(V-E_{0}\right)-h\beta_{h}\left(V-E_{0}\right) \end{array}$$ (15)

where

$$\begin{array}{ccc} \alpha_{m}\left(V\right) & = & \frac{2.5-0.1V}{\text{exp}(2.5-0.1V)-1},\;\beta_{m}\left(V\right)=4\;\text{exp}(-\frac{V}{18})\\ \alpha_{n}\left(V\right) & = & \frac{0.1-0.01V}{\text{exp}(1-0.1V)-1},\;\beta_{n}\left(V\right)=\frac{1}{8}\text{exp}(-\frac{V}{80})\\ \alpha_{h}\left(V\right) & = & 0.07\;\text{exp}(-\frac{V}{20}),\;\beta_{h}\left(V\right)=\frac{1}{\text{exp}(3-0.1V)+1}. \end{array}$$

The parameters of the Hodgkin-Huxley model are set to typical values: *C* = 1, *E*_0_ = −65, *E*_1_ = 50, *E*_2_ = −77, *E*_3_ = −54.4, *I*_stim_ = 7, and *I*_noise_ is a noisy current. Our observations of the Hodgkin-Huxley system consist of the extracellular potential approximated as

$$h\left(V,m,n,h\right)=-\frac{dV}{dt}=-(\frac{-g_{1}m^{3}h\left(V-E_{1}\right)-g_{2}n^{4}\left(V-E_{2}\right)-g_{3}\left(V-E_{3}\right)+I_{\text{stim}}+I_{\text{noise}}}{C}),$$ (16)

where 3000 ms of extracellular data sampled at rate *dt* = 0.1 are available. These observations are scaled to fit the bounds of the Fitzhugh-Nagumo model in order to prevent filter instability.

This example highlights several issues that are analogous to problems encountered in a experimental applications. For starters, the model used by the data assimilation scheme (in our case the Fitzhugh-Nagumo equations) is often a simplified representation of the experimental system producing the spike data. This results in a degree of model error, or dynamical mismatch, which in effect leads to observational bias. Additionally, the frequency of the data spikes often do not match those of the assimilation model. To help account for this discrepancy prior to assimilation, we rescale the observation time of the data to help match the spiking frequency of the model to that of the data.

**Fig 5:**
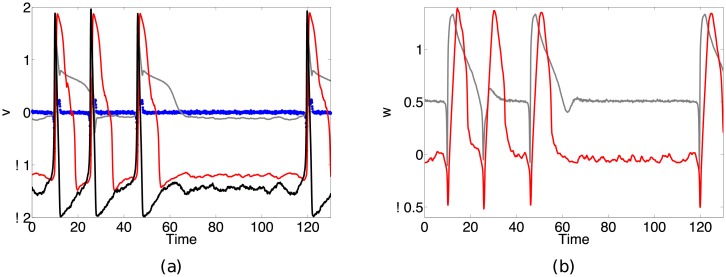
Results from assimilating extracellular Hodgkin-Huxley data (solid blue circles) to a Fitzhugh-Nagumo model. Reconstruction results for the (a) *v* and (b) *w* variables shown. The scaled Hodgkin-Huxley intracellular potential (solid black line) is shown as a reference trajectory in (a). Without bias correction (solid gray line), the filter is unable to reconcile the observational bias and as a result fails in providing a reasonable estimate of the system state. However, the bias-corrected filter (solid red line) is able to substantially improve on the state reconstruction, providing an improved estimate of the system dynamics.

Fig 5 shows the results from assimilating the extracellular Hodgkin-Huxley data (blue circles) using Eq 9 with parameters *I* = 0.5 and *τ* = 14. The scaled Hodgkin-Huxley voltage variable (solid black line) is shown in Fig 5a as a reference trajectory. Without bias correction (solid gray line), the filter is unable to estimate an accurate representation of the intracellular dynamics. Using *d* = 9 delays and *N* = 20 neighbors to reconstruct the bias, the bias-corrected filter (solid red line) is able to converge to a reasonable estimate of the intracellular variables. Note that we are not able to perfectly reconstruct the Hodgkin-Huxley voltage; this is impossible due to the discrepancy between the dynamics of the Hodgkin-Huxley data and those of the Fitzhugh-Nagumo assimilation model.

### Tracking in vivo intracelluar potential

**Fig 6:**
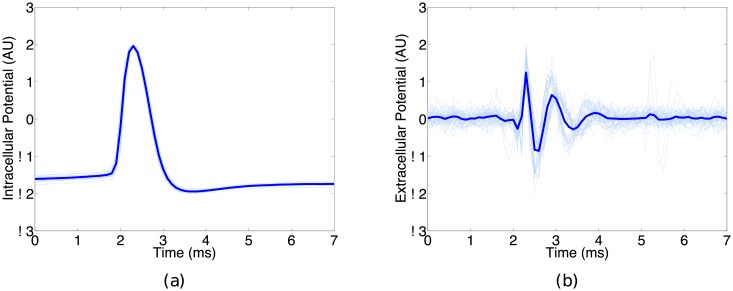
Representative waveforms (light blue traces) from recorded (a) intracellular and (b) extracellular spikes in the analyzed dataset. Dark blue lines denote the average detected waveform. Data are sampled at rate *dt* = 0.01 ms. While the authors in [4, 5] collected simultaneous intracellular and extracellular measurements, experiments usually are centered around the extracellular recordings. We therefore treat the intracellular data as a mechanism for comparison purposes, and focus on the assimilation of the readily available extracellular measurements.

We now consider the reconstruction of experimental intracellular dynamics from recorded extracellular potential. The data analyzed were collected from an experiment [3] that performed simultaneous intracellular and extracellular recordings in the CA1 region of the hippocampus of anesthetized rats [4, 5]. Fig 6 shows example waveforms, scaled to match the bounds of the assimilation model, from typical intracellular (Fig 6a) and extracellular (Fig 6b) spikes in the dataset. Light blue traces represent individual waveforms and thick solid blue line represent the average waveform. These simultaneous recordings are difficult to make and are not typical of most experimental studies, where usually only the extracellular potential is measured. As such, we treat the intracellular data strictly as a mechanism for evaluation of our assimilation results.

Our goal is to assimilate the readily available extracellular data, sampled at rate *dt* = 0.01, to the intracellular Fitzhugh-Nagumo model and reconstruct the appropriate intracellular dynamics. Note that the nature of extracellular recordings leads to measurements from several different cells, and thus in our assimilation of these observations we are in fact reconstructing the intracellular dynamics from several different neurons. Similarly to the Hodgkin-Huxley results, we don’t expect a perfect reconstruction of the intracellular potential given the amount of discrepancy between the Fitzhugh-Nagumo system and the experimental data.

**Fig 7:**
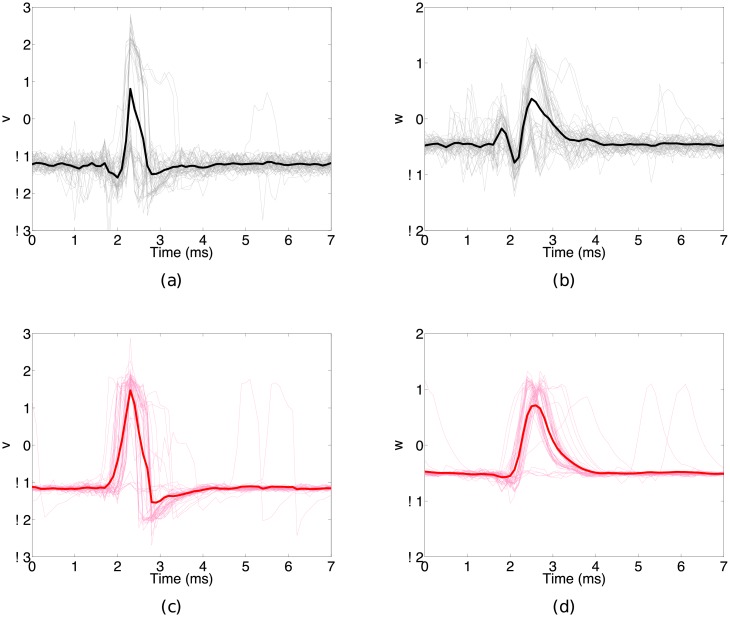
Results from assimilating the extracellular data to the Fitzhugh-Nagumo model. Thin light traces indicate individual events and the thick dark lines denote the mean waveforms averaged over individual events. Without bias correction (Fig 7 a-b), the filter is unable to compensate for the error resulting in an inaccurate estimate of the (a) intracellular potential and (b) recovery variable dynamics. When we estimate the bias and correct the observation model error (Fig 7c-d), we are able to learn the mapping from intracellular to extracellular state, and thus get an improved reconstruction of the intracellular potential and recovery dynamics, (c) and (d) respectively.

Fig 7 shows the results of estimating the intracellular dynamics given the extracellular observations. The thin light lines represent the estimated dynamics corresponding to the individual extracellular spikes shown in Fig 6b. The thick dark lines represent the average reconstructed dynamic. Without bias correction (Fig 7a and 7b), the estimate of intracellular potential and recovery variable suffers. Using *d* = 9 delays and *N* = 10 neighbors for bias reconstruction, our bias corrected filter (Fig 7c and 7d) is able to obtain an improved reconstruction of the intracellular dynamics. While the estimate is not perfect, which is to be expected since we are using an overly simplified assimilation model, we are still able to obtain a faithful representation of the intracellular potential and recovery variable.

## Conclusion

The successful implementation of data assimilation methods for estimation is dependent on an accurate mapping of the model state to the experimental measurements. This is done through application of an observation function. When this function is unknown or known with error, the observations are biased and the resulting state estimate suffers. The problem is exemplified in experimental neuroscience studies, where the relationship between extracellular potential observations and the corresponding intracellular state is complex. By leveraging a recent advance in data assimilation, we demonstrated the capability to learn this neuronal bias from available data, improving our ability to estimate intracellular neuronal state while reconciling severe model error resulting from dynamical mismatch.

As with most techniques that attempt to empirically learn a function, the resulting accuracy of the observation bias reconstruction is dependent on sufficient available data. In the neuroscience application examined here, enough spiking events must be available within the analyzed time series so that the extracellular-to-intracellular relationship can be approximated using the nearest-neighbors algorithm. Additionally, the use of any Kalman filter relies on Gaussian noise assumptions. For non-Gaussian noise processes, more sophisticated data assimilation schemes may have to be considered.

The ability to reconcile observation model error and improve on intracellular state estimation opens up several avenues for improved neuronal data analysis. As discussed in [6], the assimilation of extracellular data to intracellular models results in the filter using some of the model’s free parameters to compensate for the error. This can result in parameter estimates that have little to no interpretability. We hypothesize that our method for correcting observational bias will help improve the parameter estimation process, resulting in estimates that have greater biological meaning. Future work will examine this bias reconstruction problem in network neuroscience studies, where the goal is an accurate estimation of network connectivity given extracelluar measurements. While more sophisticated neuron models are available in the literature, one of our motivations behind choosing the Fitzhugh-Nagumo equations as our assimilation model is their scalability to network-level analysis due to the simplicity of their governing dynamics. Finally, we believe that this technique will have wide-ranging applicability to biological problems where the observation function may be known with error.
